# *In silico* prediction of drug-target interaction networks based on drug chemical structure and protein sequences

**DOI:** 10.1038/s41598-017-10724-0

**Published:** 2017-09-11

**Authors:** Zhengwei Li, Pengyong Han, Zhu-Hong You, Xiao Li, Yusen Zhang, Haiquan Yu, Ru Nie, Xing Chen

**Affiliations:** 1 School of Computer Science and Technology, China University of Mining and Technology, Xuzhou, 221116 China; 2 Cumming School of Medicine, University of Calgary, Calgary, T2N4N1 Canada; 3 The Key Laboratory of Mammal Reproductive Biology and Biotechnology, Ministry of Education, Inner Mongolia University, Hohhot, 010021 China; 4 Xinjiang Technical Institute of Physics and Chemistry, Chinese Academy of Science, Urumqi, 830011 China; 5 School of Mathematics and Statistics, Shandong University at Weihai, Weihai, 264209 China; 6 School of Information and Control Engineering, China University of Mining and Technology, Xuzhou, 21116 China

## Abstract

Analysis of drug–target interactions (DTIs) is of great importance in developing new drug candidates for known protein targets or discovering new targets for old drugs. However, the experimental approaches for identifying DTIs are expensive, laborious and challenging. In this study, we report a novel computational method for predicting DTIs using the highly discriminative information of drug-target interactions and our newly developed discriminative vector machine (DVM) classifier. More specifically, each target protein sequence is transformed as the position-specific scoring matrix (PSSM), in which the evolutionary information is retained; then the local binary pattern (LBP) operator is used to calculate the LBP histogram descriptor. For a drug molecule, a novel fingerprint representation is utilized to describe its chemical structure information representing existence of certain functional groups or fragments. When applying the proposed method to the four datasets (Enzyme, GPCR, Ion Channel and Nuclear Receptor) for predicting DTIs, we obtained good average accuracies of 93.16%, 89.37%, 91.73% and 92.22%, respectively. Furthermore, we compared the performance of the proposed model with that of the state-of-the-art SVM model and other previous methods. The achieved results demonstrate that our method is effective and robust and can be taken as a useful tool for predicting DTIs.

## Introduction

In the post-genomic era, the identification of interactions between drugs and targets plays a pivot role in developing new drug candidates for current targets and discovering new targets for old drugs. In addition, the identification of DTIs contributes to deciphering the underlying biological mechanisms and further providing great insight into various biological processes. The completion of the human genome project (HGP) and the development of molecular medicine offer great opportunity to detect interactions between drugs and targets. Although much effort has been made in recent years, few of drug candidates have been approved by the Food and Drug Administration (FDA). The main reason lies in the unacceptable toxicity and adverse side-effects for those drug candidates. Recent research definitely indicates that the interactions between drugs and certain protein targets greatly affect the toxicity or side-effects of drug candidates^1^. With the rapid increasing amount of available knowledge in biology and chemistry, a number of publicly available databases focusing on drug–target relations have been constructed, such as DrugBank^2^, SuperTarget and Matador^3^, Kyoto Encyclopedia of Genes and Genomes (KEGG)^4^, Therapeutic Target Database (TTD)^5^. These databases contain a small amount of experimentally validated interactions which are crucial for DTIs prediction and are considered as gold standards. Since the detection of DTIs by experimental methods is costly, laborious and inefficient, it is almost impossible for drug companies to carry out all experiments to identify the toxicity or side-effects of drug compound. Therefore, it is highly imperative to develop efficient and accurate computational methods to facilitate the identification of DTIs, which can provide supporting evidence for the experimental studies and therefore accelerate the discovery of new drug candidates and targets.

So far, a number of *in silico* methods have been developed to address the issues of DTI prediction^6–12^. There are two main traditional computational methods, namely ligand-based and receptor-based approach. The ligand-based virtual screening method utilizes chemical structure similarity to predict DTIs. For instance, Keiser *et al*. adopted chemical 2D structural similarity of ligands to predict new molecular targets^8^. Campillos *et al*. employed phenotypic side-effect similarities to identify the interactions between drugs and targets^9^. The ligand-based approach, however, may not perform well for target proteins with a small number of known ligands. Receptor-based method like reverse docking has also been applied in DTI prediction when drug molecule and target protein bind each other^10–12^. However, this kind of method could not be applied to targets whose 3D structures are unknown. Therefore, the efficient computational methods directly based on protein sequence rather than 3D structure of protein appear to be useful for predicting DTIs.

Recently, a variety of computational methods based on machine learning have been proposed to predict DTIs by building a classification model treating each drug-target pair as one sample^13–18^. These studies are mainly based on the assumption that similar drug molecules are likely to interact with similar target proteins. The drug-target pairs with known interaction are labeled as positive samples while randomly connected pairs (non-interacting) are treated as negative ones. Each sample is a concatenation of drug feature vector and protein feature vector. Francisco *et al*. proposed a multi-target QSAR model to predict DTIs by calculating 2D molecular descriptors for drug feature extraction^14^. Mei *et al*. proposed their BLM-NII algorithm to predict new target probability of a specified drug which is highly reliable in predicting DTIs^15^. Chen *et al*. employed a machine learning based approach to identify drug target groups by integrating the compound information of chemical–chemical similarities, chemical–chemical connections and chemical–protein connections^16^.

In this study, we attempt to formulate the DTIs as an extended structure–activity relationship (SAR) classification problem. The interactions between drugs and their targets can be considered as “activity” properties, which are largely dependent on the structural information from both drug molecules and target proteins. We represent drugs by their substructure fingerprints representing existence of certain functional groups or fragments, employ local binary pattern (LBP) to transform target protein sequence data and apply principal component analysis (PCA) to the connected feature vector to reduce the impact of noises. Then, our newly developed discriminative vector machine (DVM) classifier is employed in the classification for the four pharmaceutically gold targets: *Enzyme*, *GPCR*, *Ion Channel and Nuclear Receptor*. DVM is a probably approximately correct (PAC) learning algorithm which can reduce the error caused by generalization and has strong robustness^19^. The achieved results indicate that our method is effective and robust and can be taken as a useful tool for further studies of DTIs.

## Results and Discussion

### Evaluation metrics

To evaluate the performance of related approaches, four evaluation metrics, including precision (*Pre*), accuracy (*Acc*), sensitivity (*Sen*), and Matthews’s correlation coefficient (*MCC*), are calculated accordingly. Their corresponding calculating formulas are as follows:

1 $$Pre=\frac{TP}{TP+FP}$$

2 $$Sen=\frac{TP}{TP+FN}$$

3 $$Acc=\frac{TP+TN}{TP+FP+TN+FN}$$

4 $$MCC=\frac{(TP\times TN)-(FP\times FN)}{\sqrt{(TP+FN)\times (TN+FP)\times (TP+FP)\times (TN+FN)}}$$

where ${TP}$ represents the number of interacting drug-target pairs predicted correctly (i.e., true positive) while ${TN}$ stands for the number of non-interacting drug-target pairs predicted correctly (i.e., true negative). Similarly, ${FP}$ is the number of non-interacting drug-target pairs falsely predicted to be interacting drug-target pairs (i.e., false positive), and ${FN}$ denotes the number of interacting drug-target pairs falsely predicted to be non-interacting drug-target pairs (i.e., false negative). Additionally, receiver operating characteristic (ROC) curves are calculated for evaluating the performance of the proposed method and SVM-based method. A model with no prediction ability would yield the diagonal line. The closer the ROC area is to 1, the higher the prediction ability of model is. To summarize ROC curve in a numerical way, the area under an ROC curve (AUC) is calculated accordingly.

### Results of proposed method on the four gold datasets

In this study, to reduce data dependence and avoid overfitting of the proposed method, five-fold cross validation was employed as testing strategy. Specifically, each dataset (*Enzyme*, *GPCR*, *Ion Channel and Nuclear Receptor*) was randomly split into five parts of roughly equal size of which four parts of them served to train the DVM model, and the remaining part was set aside for testing in turn. The whole process is repeated five times and five prediction models were constructed, tested and evaluated separately. To be fair, the parameters of DVM classifier were set to the same on all the four datasets.

**Figure 1:**
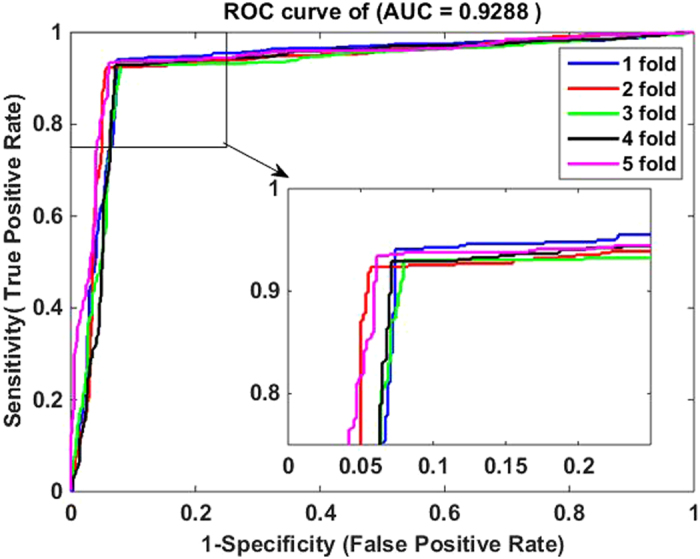
ROC curves by our method on the Enzyme dataset.

**Figure 2:**
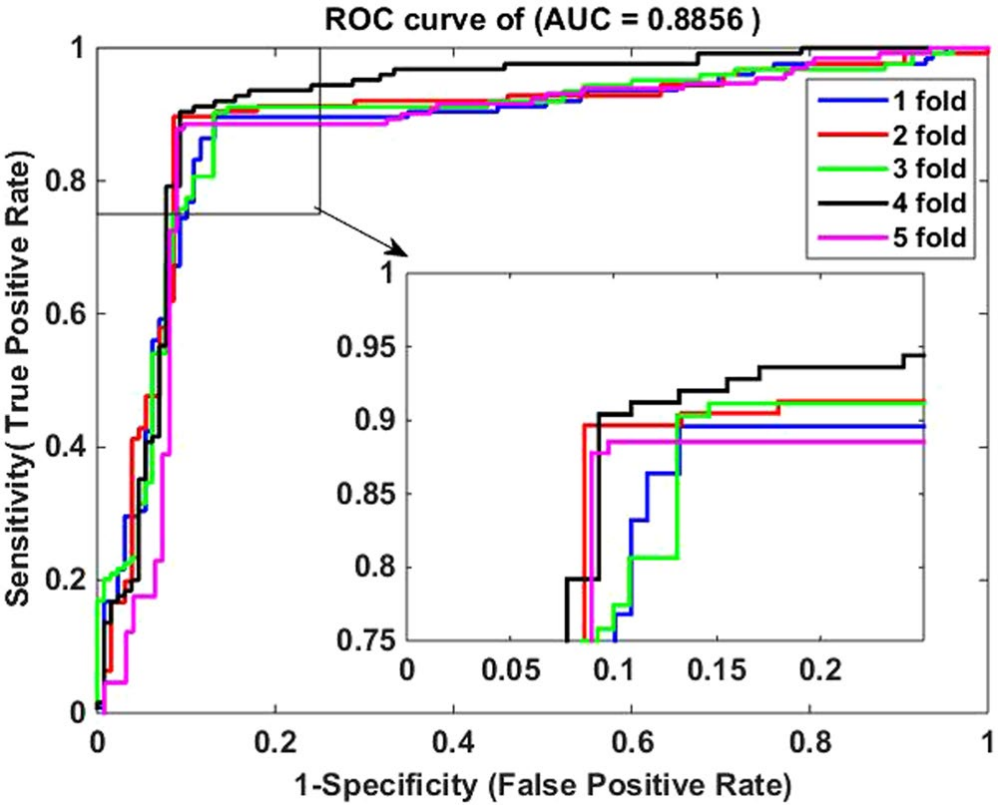
ROC curves by our method on the GPCR dataset.

**Figure 3:**
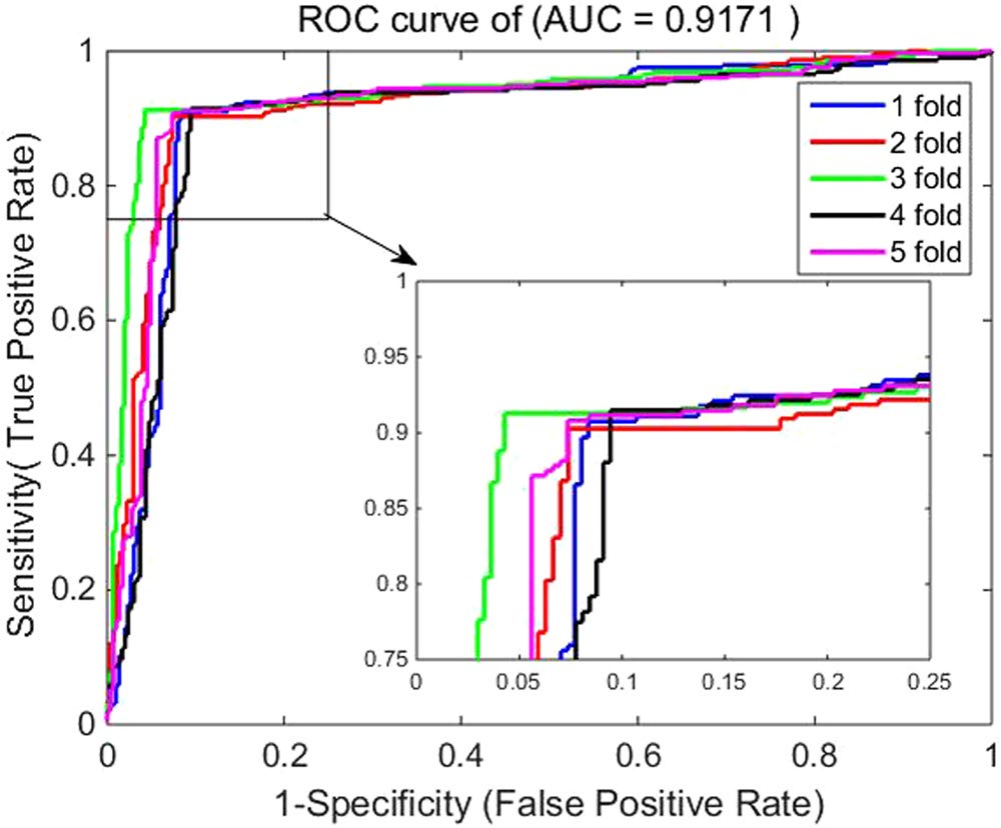
ROC curves by our method on the Icon Channel dataset.

**Figure 4:**
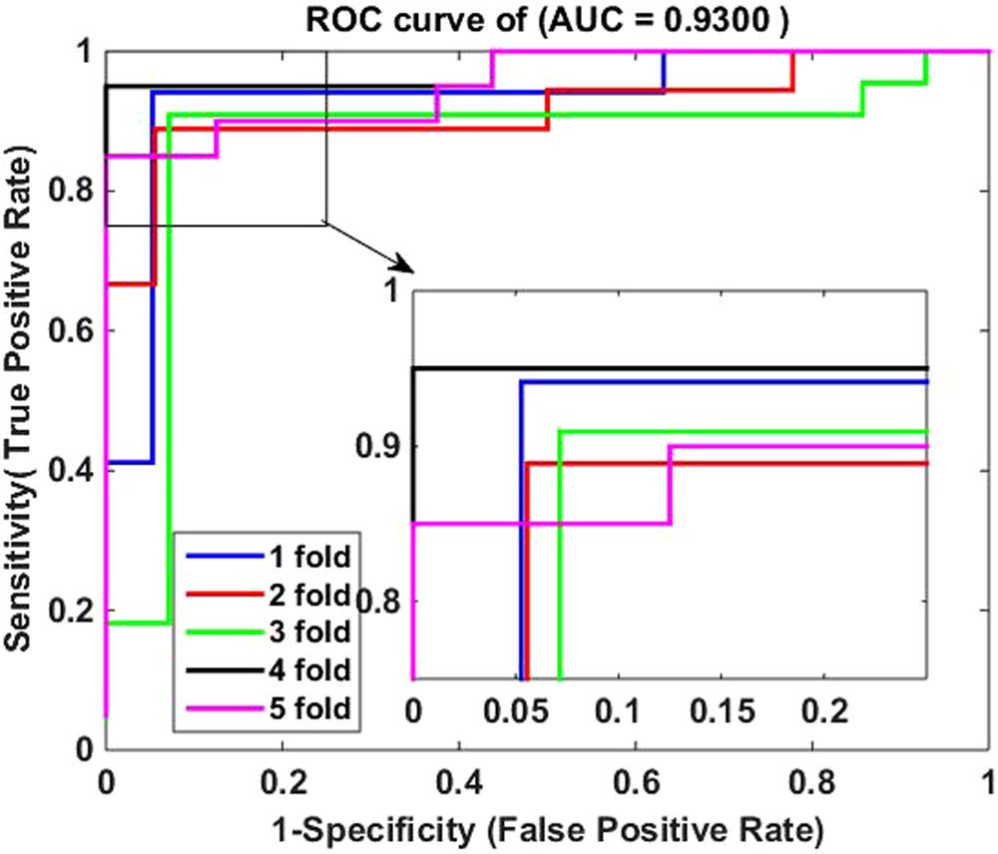
ROC curves by our method on the Nuclear Receptor dataset.

The five-fold cross validation results of the proposed method on all the four benchmark datasets are listed in Tables 1–4. When applying the proposed method to the *Enzyme* dataset, we obtain the best prediction results of average precision (Pre), accuracy (Acc), sensitivity (Sen), Matthews’s correlation coefficient (MCC) and area under ROC curve (AUC) of 93.18%, 93.16%, 92.90%, 86.32% and 92.88%, respectively, and their standard deviations are 0.64%, 0.43%, 1.19%, 0.88% and 0.87%, respectively (see Table 1). On the *GPCR* dataset, our method yields the average precision, accuracy, sensitivity, MCC and AUC of 89.40%, 89.37%, 89.27%, 78.77% and 88.56%, respectively, and their standard deviations are 1.10%, 1.21%, 2.30%, 2.41% and 1.74%, respectively (see Table 2). Similarly, it can be seen from Table 3 that the average precision, accuracy, sensitivity, MCC and AUC on the *Icon Channel* dataset reach 90.90%, 91.73%, 92.65%, 83.47% and 91.71%, respectively, and the corresponding standard deviations are 0.47%, 0.96%, 1.74%, 1.94% and 1.36%, respectively. In Table 4, the averages of precision, accuracy, sensitivity, MCC and AUC on the *Nuclear Receptor* dataset are 88.67%, 92.22%, 96.62%, 84.80% and 93.00% respectively. However, their standard deviations are 4.43%, 2.32%, 3.13%, 4.60% and 4.19%, respectively, which are the highest values in the four tables. The possible reason for such results is that the number of samples in *the Nuclear Receptor* dataset is only 90, relatively less than that of other datasets. The receiver operating characteristic (ROC) curves performed by our method on the four benchmark datasets are illustrated in Figures 1–4.

**Table 1:** Five-fold cross validation results by our method on the *Enzyme* dataset.

Test set	Pre (%)	Acc (%)	Sen (%)	MCC (%)	AUC (%)
1	94.10	93.33	92.12	86.67	93.21
2	92.37	93.33	94.29	86.69	92.94
3	93.06	92.56	92.28	85.13	91.93
4	92.92	92.91	91.73	85.74	92.22
5	93.46	93.68	94.09	87.35	94.13
Average	**93.18 ± 0.64**	**93.16 ± 0.43**	**92.90 ± 1.19**	**86.32 ± 0.88**	**92.88 ± 0.87**

**Table 2:** Five-fold cross validation results by our method on the *GPCR* dataset.

Test set	Pre (%)	Acc (%)	Sen (%)	MCC (%)	AUC (%)
1	88.80	87.80	86.72	75.61	87.73
2	89.68	90.55	91.13	81.11	88.71
3	90.32	88.58	86.82	77.23	88.31
4	90.40	90.55	90.40	81.10	91.37
5	87.79	89.37	91.27	78.81	86.69
Average	**89.40 ± 1.10**	**89.37 ± 1.21**	**89.27 ± 2.30**	**78.77 ± 2.41**	**88.56 ± 1.74**

**Table 3:** Five-fold cross validation results by our method on the *Icon Channel* dataset.

Test set	Pre (%)	Acc (%)	Sen (%)	MCC (%)	AUC (%)
1	90.72	91.19	91.35	82.37	90.89
2	90.28	91.36	93.51	82.71	91.91
3	91.26	93.39	94.91	86.81	93.75
4	91.47	91.02	90.54	82.04	90.14
5	90.79	91.69	92.93	83.41	91.85
Average	**90.90 ± 0.47**	**91.73 ± 0.96**	**92.65 ± 1.74**	**83.47 ± 1.94**	**91.71 ± 1.36**

**Table 4:** Five-fold cross validation results by our method on the *Nuclear Receptor* dataset.

Test set	Prec (%)	Accu (%)	Sen (%)	MCC (%)	AUC (%)
1	94.12	94.44	94.12	88.85	93.50
2	83.33	88.89	93.75	78.26	91.67
3	90.91	91.67	95.24	82.83	86.69
4	90.00	94.44	100.00	89.44	97.81
5	85.00	91.67	100.00	84.60	95.31
Average	**88.67 ± 4.43**	**92.22 ± 2.32**	**96.62 ± 3.13**	**84.80 ± 4.60**	**93.00 ± 4.19**

From Tables 1–4, we can observe that the powerful DVM-based prediction model combined with LBP histogram protein descriptor and drug substructure fingerprints is accurate, effective and robust for predicting drug-target interactions. We owe the good performance of the proposed method to the choice of effective feature extraction method and the powerful DVM classifier. In addition, the LBP histogram descriptors of target proteins not only retain the sufficient evolutionary information of amino acids, but also differentiate amino acids effectively while substructure fingerprints also contain highly discriminative information of drugs.

To validate the performance of our unbiased method not strongly related to the selection of negative samples, without loss of generality, we also carried out additional five-fold cross validation with five different negative training samples (non-interacting) randomly selected from the *GPCR and drug molecules* dataset. As shown in Table 5, although the obtained results on different negative training samples are slight different, these results are consistency in general and the average precision, accuracy, sensitivity, MCC and AUC are all higher than 89%, 88%, 87%,77% and 86%, respectively, which further demonstrate that our approach for selecting negative samples in this study is appropriate for assessing prediction performance.

**Table 5:** Comparisons of five-fold cross validation prediction performance using five different randomly selected negative training samples on the *GPCR* dataset.

Negative Samples	Prec (%)	Accu (%)	Sen (%)	MCC (%)	AUC (%)
1	89.65 ± 2.20	88.61 ± 0.95	89.50 ± 1.62	79.21 ± 1.92	89.84 ± 1.26
2	89.13 ± 2.04	88.98 ± 2.25	87.87 ± 2.64	77.98 ± 2.59	86.67 ± 2.15
3	90.39 ± 2.33	90.16 ± 1.72	89.91 ± 1.39	80.35 ± 1.41	91.14 ± 1.64
4	91.68 ± 2.89	90.08 ± 2.51	88.36 ± 2.74	80.50 ± 2.03	90.04 ± 2.99
5	89.74 ± 2.69	89.37 ± 1.95	89.06 ± 2.98	78.88 ± 2.15	89.76 ± 2.43

### Comparisons between discriminative vector machine and support vector machine

To further evaluate the proposed method, the state-of-the-art support vector machine (SVM) classifier was constructed accordingly. Here we used LIBSVM toolbox as SVM classifier to carry out the prediction of DTIs. To be fair, the two methods adopted the same feature data on all the four gold dataset. A general grid search scheme was used to optimize LIBSVM’s two parameters (regularization parameter $C$, kernel width parameter $\gamma$) and they ($C$, $\gamma$) were at last tuned to 0.5 and 0.7, respectively. Additionally, Gaussian function was chosen as the kernel function. For the DVM and SVM classifiers, all the input feature vectors were normalized in the range of [0, 1].

**Figure 5:**
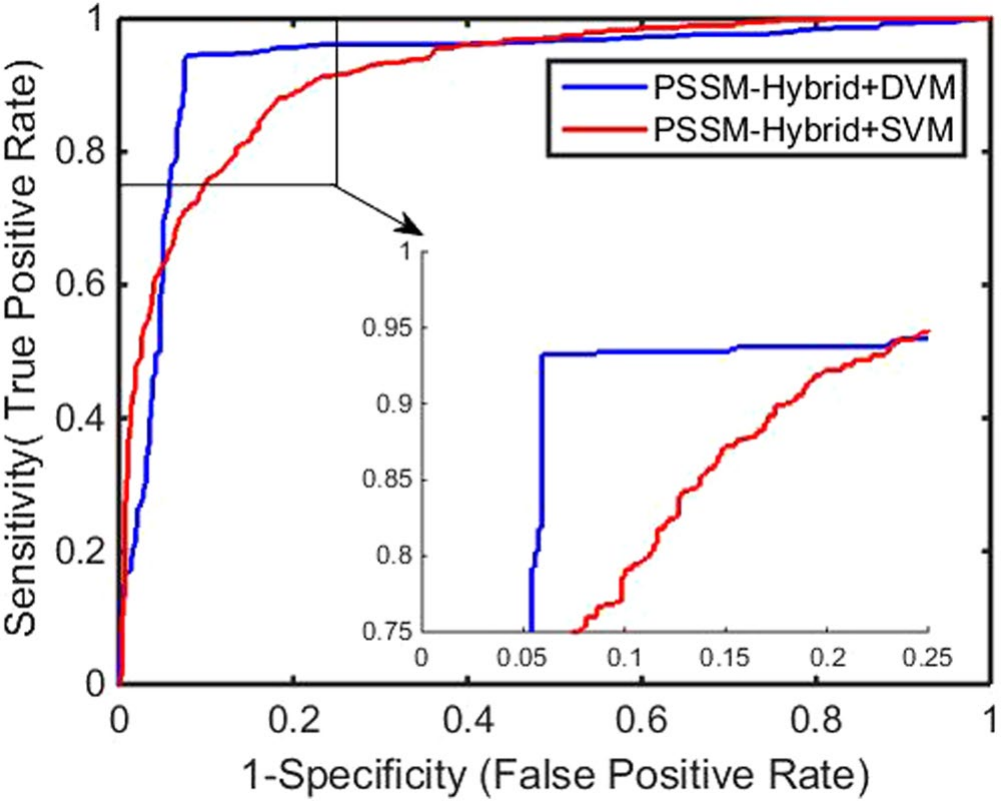
Comparison of ROC curves between DVM and SVM on the Enzyme dataset.

**Figure 6:**
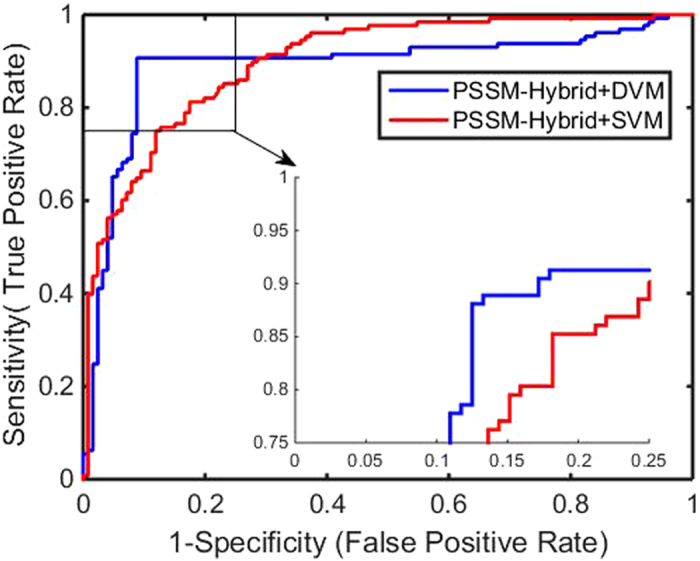
Comparison of ROC curves between DVM and SVM on the GPCR dataset.

**Figure 7:**
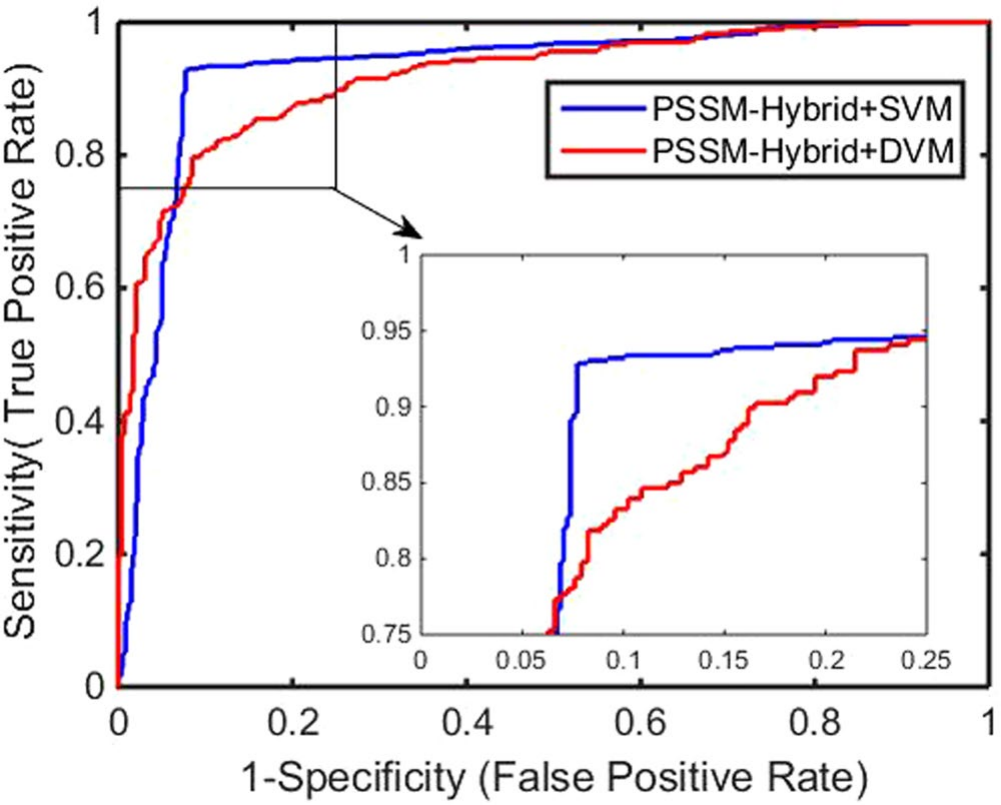
Comparison of ROC curves between DVM and SVM on the Ion Channel dataset.

**Figure 8:**
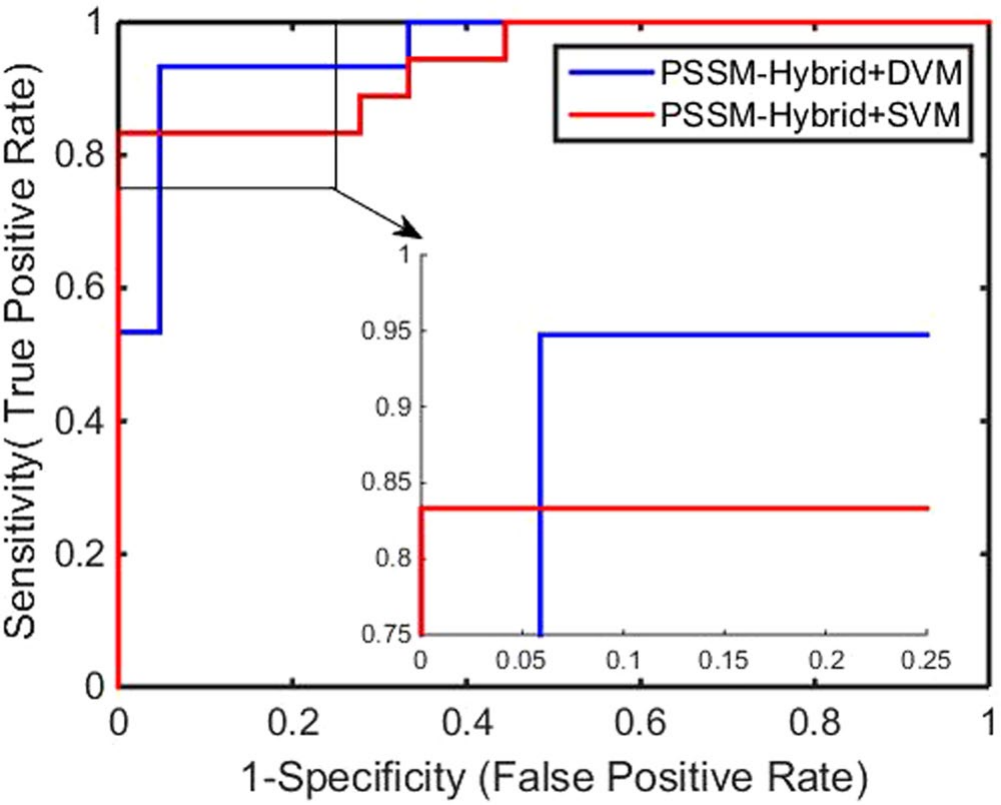
Comparison of ROC curves between DVM and SVM on the Nuclear Receptor dataset.

The predictive results of the two methods are summarized in Tables 6–9 and the corresponding ROC curves are illustrated in Figures 5–8. It can be drawn from these tables and figures that the achieved results hold nearly the same varying tendency. Taking the *Ion Channel* dataset as an example, the averages of Pre, Acc, Sen, MCC and AUC of SVM reach 85.12%, 85.59%, 86.24%, 71.24% and 85.89%, respectively, significantly lower than those by DVM, which are 90.90%, 91.73%, 92.65%, 83.47% and 91.71%, respectively. Similarly, the majority of their standard deviations of SVM are also higher than those of DVM. Additionally, as shown in Figures 5–8, the ROC curves of the DVM-based prediction model are superior to those of the SVM-based classifier, which suggests that DVM with the same feature descriptors performs better than SVM in general. There are two possible reasons for such results. (1) Based on k nearest neighbors (kNNs), robust M-estimator and manifold regularization, DVM reduces the influence of outliers and overcomes the weakness of the kernel function to meet the Mercer condition. (2) Although there are three parameters (β, γ, and θ) in DVM model, those parameters slightly affect its performance and they are more easily tuned than those of SVM.

**Table 6:** Five-fold cross validation results on the *Enzyme* dataset of DVM and SVM.

Model	Testing Set	Pre (%)	Acc (%)	Sen (%)	MCC (%)	AUC (%)
SVM	1	90.24	90.68	91.31	81.37	90.70
	2	88.64	89.15	89.71	78.30	88.47
	3	89.85	90.00	90.60	79.99	89.96
	4	90.39	91.71	92.78	83.44	91.37
	5	90.81	89.68	88.51	79.38	89.26
	Average	**89.99 ± 0.74**	**90.24 ± 0.88**	**90.58 ± 1.44**	**80.50 ± 1.78**	**89.95 ± 1.02**
DVM	1	94.10	93.33	92.12	86.67	93.21
	2	92.37	93.33	94.29	86.69	92.94
	3	93.06	92.56	92.28	85.13	91.93
	4	92.92	92.91	91.73	85.74	92.22
	5	93.46	93.68	94.09	87.35	94.13
	Average	**93.18 ± 0.64**	**93.16 ± 0.43**	**92.90 ± 1.19**	**86.32 ± 0.88**	**92.88 ± 0.87**

**Table 7:** Five-fold cross validation results on the *GPCR* dataset of DVM and SVM.

Model	Testing Set	Pre (%)	Acc (%)	Sen (%)	MCC (%)	AUC (%)
SVM	1	85.04	85.43	85.71	70.87	84.92
	2	85.82	83.86	83.94	67.59	84.86
	3	84.03	84.25	82.64	68.42	85.43
	4	83.87	85.43	85.95	70.85	87.18
	5	87.41	88.58	90.77	77.19	88.64
	Average	**85.23 ± 1.30**	**85.51 ± 1.66**	**85.80 ± 2.76**	**70.98 ± 3.37**	**86.21 ± 1.48**
DVM	1	88.80	87.80	86.72	75.61	87.73
	2	89.68	90.55	91.13	81.11	88.71
	3	90.32	88.58	86.82	77.23	88.31
	4	90.40	90.55	90.40	81.10	91.37
	5	87.79	89.37	91.27	78.81	86.69
	Average	**89.40 ± 1.10**	**89.37 ± 1.21**	**89.27 ± 2.30**	**78.77 ± 2.41**	**88.56 ± 1.74**

**Table 8:** Five-fold cross validation results on the *Ion Channel* dataset of DVM and SVM.

Model	Testing Set	Pre (%)	Acc (%)	Sen (%)	MCC (%)	AUC (%)
SVM	1	84.46	85.93	87.11	71.90	85.40
	2	85.71	84.58	83.11	69.19	85.19
	3	87.75	86.10	85.48	72.20	86.11
	4	83.55	84.75	86.39	69.53	85.48
	5	84.11	86.61	89.12	73.36	87.28
	Average	**85.12 ± 1.67**	**85.59 ± 0.89**	**86.24 ± 2.21**	**71.24 ± 1.80**	**85.89 ± 0.85**
DVM	1	90.72	91.19	91.35	82.37	90.89
	2	90.28	91.36	93.51	82.71	91.91
	3	91.26	93.39	94.91	86.81	93.75
	4	91.47	91.02	90.54	82.04	90.14
	5	90.79	91.69	92.93	83.41	91.85
	Average	**90.90 ± 0.47**	**91.73 ± 0.96**	**92.65 ± 1.74**	**83.47 ± 1.94**	**91.71 ± 1.36**

**Table 9:** Five-fold cross validation results on the *Nuclear Receptor*dataset of DVM and SVM.

Model	Testing Set	Pre (%)	Acc (%)	Sen (%)	MCC (%)	AUC (%)
SVM	1	83.33	83.33	83.33	66.67	84.26
	2	80.00	86.11	94.12	73.41	86.38
	3	86.67	75.00	65.00	52.92	71.56
	4	76.47	75.00	72.22	50.08	73.46
	5	82.35	83.33	82.35	66.56	83.59
	Average	**81.76 ± 3.41**	**80.55 ± 4.65**	**79.40 ± 10.00**	**61.93 ± 8.91**	**79.85 ± 6.09**
DVM	1	94.12	94.44	94.12	88.85	93.50
	2	83.33	88.89	93.75	78.26	91.67
	3	90.91	91.67	95.24	82.83	86.69
	4	90.00	94.44	100.00	89.44	97.81
	5	85.00	91.67	100.00	84.60	95.31
	Average	**88.67 ± 4.43**	**92.22 ± 2.32**	**96.62 ± 3.13**	**84.80 ± 4.60**	**93.00 ± 4.19**

### Comparison with previous studies

As mentioned before, there are a variety of computational methods for predicting drug-target interactions. To further illustrate the effectiveness of the proposed approach, we compared its performance with other published methods which adopted the same five-fold cross validation framework and were based on the same four datasets. Table 10 lists the average AUC (area under the receiver operating curve) values for the method by Jian-yu Shen *et al*.^20^, NetCBP^21^, the method by Yanamishi *et al*.^22^, KBMF2K^7^, and our proposed method. It can be observed that the proposed method has an obvious improvement in the prediction performance for DTIs in term of AUC. The average growths of our result to the best result of four previous methods on the datasets of *Enzyme*, *GPCR*, *Ion Channel* and *Nuclear Receptor* are 9.92%, 1.21%, 13.08% and 6.77%, respectively. The high predictive performance of the proposed method may attribute to the novel feature extraction method which extracts highly discriminative information of target proteins and drug molecules, and the use of DVM classifier which has been demonstrated to be robust and powerful.

**Table 10:** Prediction performances of NetCBP^21^, Yamanishi *et al*.^22^, KBMF2K^7^, and our method on the four benchmark datasets in terms of average AUC.

Dataset	Our method	Shen *et al*. (2015)	NetCBP	Yamanishi *et al*.(2010)	KBMF2K
*Enzyme*	**0.9288 ± 0.0087**	0.812	0.8251	0.845	0.832
*GPCR*	**0.8856 ± 0.0174**	0.875	0.8235	0.812	0.857
*Icon Channel*	**0.9171 ± 0.0136**	0.811	0.8034	0.731	0.799
*Nuclear Receptor*	**0.9300 ± 0.0419**	0.871	0.8394	0.830	0.824

## Conclusion

In the post-genomic era, study of drug-target interactions is very important in developing new drug candidates for current targets and discovering new targets for old drugs. However, experimental methods for identifying DTIs are time-consuming, costly and challenging even nowadays. In this work, we propose a novel computational method for predicting DTIs which makes the best of the substructure fingerprints of drug molecules and the sequence information of target proteins. Additionally, the biological evolutionary information of protein is also taken into account during the process of feature extraction. When applied to the four benchmark datasets (*Enzyme*, *GPCR*, *Ion Channel and Nuclear Receptor*), the proposed method achieves average accuracies of 93.16%, 89.37%, 91.73% and 92.22%, respectively. To further evaluate the performance of the proposed method, it is compared with SVM-based model and other previous approaches. The achieved results show that our proposed method is highly competitive and can be taken as a powerful tool for predicting drug-target interactions.

## Materials and Methods

### Gold standard datasets

In this study, we evaluate the predictive method of DTIs on four gold benchmark datasets, namely *Enzyme, GPCR, Ion Channel and Nuclear Receptor*, which are from KEGG BRITE^23^, SuperTarget & Matador^3^, DrugBank^24^ and BRENDA^25^, respectively. As shown in Table 11 the number of known drugs targeting *Enzyme*, *GPCR*, *Ion Channel and Nuclear Receptor*, are 445, 223, 210 and 54, respectively, and the number of known protein targeted by these drugs are 664, 95, 204 and 26, respectively. The number of known interactions between these drugs and targets are 2926, 635, 1476 and 90, respectively. Therefore, the total interacting pairs of drug-target are 5127 and they are then employed to build the positive samples in the cross-validation experiments.

**Table 11:** The four drug–target interaction datasets.

Dataset	drug compounds	target proteins	Interactions
*Enzyme*	445	664	2926
*GPCR*	223	95	635
*Ion Channel*	210	204	1476
*Nuclear Receptor*	54	26	90

In general, drug-target interactions network is usually formulated as a bipartite graph where drug molecules and target proteins are nodes and the known drug–target interactions are edges between these nodes. Compared with a fully connected bipartite graph, the number of initial edges is extremely small. Take *ion channel* dataset as an example, its corresponding bipartite graph has up to 210 × 204 = 42840 edges. However, there are only 1476 initial connections which is significantly less than the number of possible negative samples (42840–1476 = 42364). To correct the bias caused by the imbalance samples, we randomly selected the non-interacting drug-target pairs (as negative samples) with the same number of the interacting drug-target pairs (as positive samples). As a matter of fact, such a set of negative samples generated randomly may contain very few drug-target pairs interacting really; nevertheless, in view of the large scale of DTIs, the number of real interactions pairs possibly collected in negative sets is very small.

### Representation of drug molecules and target proteins

#### Representation of drug molecules

A variety of descriptors for encoding drug compounds have been proposed, including topological, constitutional, geometrical and quantum chemical properties etc. Additionally, recent studies indicate that drug compounds can also be effectively represented by the molecular substructure fingerprints^26, 27^. Substructure fingerprints can directly encode molecular structure information in binary bits which denote the absence or presence of specific substructures of a given drug molecule. If a substructure exists in a given drug molecule, the corresponding bit in fingerprint is assigned to 1, or else to 0. Although the substructure fingerprint divides the whole molecule into a number of fragments, it has the ability to retain highly discriminative structural information of drug molecules. In addition, it does not require the 3D conformation of drug compound and thereby does not cause error accumulation. The substructure fingerprints sets adopted in this study are collected from the PubChem system. The drug fingerprints record the information of mostly common 615 substructures and therefore the length of feature vector of drug molecule is 615.

#### Representation of target proteins

Effective protein descriptors can provide highly discriminatory nature for identifying DTIs and thus boost the performance of prediction model. Up to now, there are many feature descriptors proposed for protein sequences. Most of these descriptors are based on the position-specific scoring matrix (PSSM) of protein sequences. PSSM is a representation of a protein sequence which provides the probability of any given amino acid occurring at a particular position and carries the evolutionary information of the sequence^28^. In this study, we adopt the position specific iterated BLAST (PSI-BLAST) tool to create PSSMs for all target protein sequences, via 3 iterations setting the E-value cutoff at 0.001 for the query protein sequence against multiple sequence alignment. The PSSM of a query protein sequence can be expressed as

5 $$P=\{{P}_{i}^{j}\},i=1,2,\ldots ,L,j=1,2,\ldots ,20$$

where $L$ is the length of the protein sequence and 20 denotes the 20 standard amino acids; ${P}_{i}^{j}$ is the score for the ${jth}$ amino acid in the ${ith}$ position of the given protein sequence^29^.

Local binary pattern (LBP)^30^ is a powerful operator for image description that is based on the signs of differences of neighboring pixels. Despite its simplicity, LBP is very descriptive and has been successfully applied to a wide variety of different tasks. The original version of the descriptor labels the pixels by threshold the 3 × 3 neighborhood of each pixel with the center value and summing the threshold values weighted by 2 to the power of $i$. Given a pixel of an image, an LBP operator is calculated as follow:

6 $$LB{P}_{P,R}=\sum _{i=0}^{P-1}s({v}_{i}-{v}_{c}){2}^{i},s(x)=\{\begin{array}{c}0,\,x < 0\\ 1,x\ge 0\end{array}$$

where ${v}_{c}$ is the value of central pixel, ${v}_{i}$ is the value of its neighbors, $P$ represents the total number of sampling points and $R$ is the radius of the neighborhood. Furthermore, two extensions of original operator are proposed by Ojala *et al*.^30^. (1) Different sizes of neighborhood were employed to retain discriminative features at different scales. (2) Uniform patterns were proposed to use a small subset of ${2}^{P}$ patterns, which contain at most two bitwise transitions from 0 to 1 or vice versa. After labeling an image with a LBP operator, a histogram of the labeled image can be defined as

7 $${H}_{i}=\sum _{x,y}I(f(x,y)=i),i=1,\ldots ,S$$

where $S$ is the number of different labels produced by LBP operator and $I(\gamma )$ is 1 if $\gamma$ is true and 0 otherwise. In this work, each PSSM matrix of a protein sequence is treated as an image and the number of neighbors is set to 8. After a PSSM matrix is processed by LBP histogram operator, a corresponding 256-dimentioanl feature vector is formed accordingly.

#### feature reduction by PCA

Principal component analysis (PCA) is a statistical method that uses an orthogonal transformation to convert a set of observations of possibly correlated variables into a set of values of linearly uncorrelated variables called principal components. To reduce computing load and the influence of noise, PCA is introduced to extract the most discriminatory low-dimensional features of both drugs and target proteins. The obtained compact representations of drug compounds and target proteins are then employed to identify their interactions.

### Discriminative Vector Machine

Classification is a fundamental issue in pattern recognition field and there are a wide variety of classification algorithms. In this study, our newly developed discriminative vector machine (DVM) classifier is adopted for classification prediction. DVM is a probably approximately correct (PAC) learning model which can reduce error accumulation and has strong robustness^19^. Given a test sample $y$, the first step of DVM is to find its top $k$ nearest neighbors (kNNs) to suppress the effect of outliers. The kNNs of $y$ can be expressed by *X*_*k*_ = [*x*_1_, *x*_2_, *…*, *x*_*k*_], where ${x}_{i}$ is the ${ith}$ nearest neighbor. For convenience, ${X}_{k}$ can be also represented as *X*_*k*_ = [*x*_*k*,1_, *x*_*k*,2_, …, *x*_*k*,*c*_], where ${x}_{k,j}$ comes from the ${jth}$ class. Then the objective of DVM is to solve the following minimization problem:

8 $${}_{{\beta }_{k}}{}^{min}\,\sum _{i=1}^{d}\varnothing ({(y-{X}_{k}{\beta }_{k})}_{i})+\delta \Vert {\beta }_{k}\Vert +\gamma \sum _{p=1}^{k}\sum _{q=1}^{k}{w}_{pq}{({\beta }_{k}^{p}-{\beta }_{k}^{q})}^{2}$$

where ${(y-{X}_{k}{\beta }_{k})}_{i}$ is the $ith$ element of $y-{X}_{k}{\beta }_{k}$ and ${\beta }_{k}$ has the form of $[{\beta }_{k}^{1},\,{\beta }_{k}^{2},\,\ldots ,{\beta }_{k}^{k}]$ or $[{\beta }_{k,1},{\beta }_{k,2},\ldots ,{\beta }_{k,c}]$, where ${\beta }_{k,i}$ is the coefficient from the ${ith}$ class. ${\rm{\varnothing }}$ is a M-estimator used to improve the robustness of DVM. There are many robust estimators like Welsch M-estimator, MBA (Median Ball Algorithm) estimator and Cauchy M-estimator^31^. In this study, a robust Welsch M-estimator $(\varnothing (x)=(1/2)(1-{\exp }(-{x}^{2}))$) is adopted to attenuate error accumulation so that outliers would have less impact on prediction. ||*β*_*k*_|| is a norm of *β*_*k*_ and the corresponding *l*2-norm is adopted accordingly. The last section of equation (8) is the manifold regularization where ${w}_{{pq}}$ is the similarity between the ${pth}$ and the ${qth}$ nearest neighbor (NN) of $y$. In this work, ${w}_{{pq}}$ is defined as the cosine distance between the ${pth}$ and the ${qth}$ NN of $y$. Thus the corresponding Laplacian matrix L can be depicted as

9 $$L=D-W$$

where $W$ is the similarity matrix whose element is $\,{w}_{pq}(p=1,\,2,\ldots ,k;\,q=1,\,2,\ldots ,k),$ $D$ is a diagonal matrix whose $ith$ element ${d}_{i}$ is the sum of $\,{w}_{iq}(q=1,\,2,\ldots ,k)$. According to equation (9), the last section of equation (8) can be represented as $\gamma {\beta }_{k}^{T}L{\beta }_{k}$. Construct a diagonal matrix *P* = diag(*p*_*i*_) whose element $\,{p}_{i}(i=1,\,2,\ldots ,d)$ is

10 $${p}_{i}={e}^{-\frac{{({(y-{X}_{k}{\beta }_{k})}_{i})}^{2}}{{\sigma }^{2}}}\,$$

where $\sigma$ is the kernel size which can be calculated by:

11 $$\sigma =\sqrt{(\theta \times {(y-{X}_{k}{\beta }_{k})}^{T}\times (y-{X}_{k}{\beta }_{k})/d)}$$

where *θ* is a constant to suppress the effect of outliers. In this work, it is set to 1.0 as in the literature^32^. Based on the equations (9), (10) and (11), the minimization of the equation (8) can be represented as

12 $${ar}{{g}}_{{\beta }_{k}}^{min}{(y-{X}_{k}{\beta }_{k})}^{T}P(y-{X}_{k}{\beta }_{k})+\delta {\beta }_{k2}^{2}+\gamma {\beta }_{k}^{T}L{\beta }_{k}$$

According to the theory of half-quadratic minimization, the global solution ${\beta }_{k}$ of equation (12) can be addressed by:

13 $${\beta }_{k}={({X}_{k}^{T}P{X}_{k}+\delta I+\gamma L)}^{-1}{X}_{k}^{T}Py$$

After the related coefficients for each class are calculated, the test sample $y$ can be identified as the ${ith}$ class if the residual $\Vert y-{X}_{{ki}}{\beta }_{{ki}}\Vert$ is minimal.

14 $${{\rm{R}}}_{{\rm{i}}}={}_{i\,}{}^{{\rm{\min }}}\Vert {\rm{y}}-{{\rm{X}}}_{{\rm{ki}}}{{\rm{\beta }}}_{{\rm{ki}}}\Vert ,{\rm{i}}=1,2,\ldots ,c\,$$

In this work, there are two classes in total to be identified: non-interacting drug-target pair (class 1) and interacting drug-target pair (class 2). If ${R}_{1}$ are less than ${R}_{2}$, the sample $y$ will be classified as non-interacting drug-target pair (class 1), otherwise as interacting drug-target pair (class 2). For three free parameters ($\delta$, $\gamma$, $\theta$) of the DVM model, it is time-consuming to directly search their optimal values. It is gratifying that DVM model is so stable that all these parameters only affect its performance slightly if they are set in the feasible ranges. Based on the above knowledge and through grid search, the parameters $\delta$ and $\gamma$ are assigned as 1*E*-3 and 1*E*-4 respectively. Just as described before, $\theta$ is a constant and is set to 1 throughout the whole process. Actually, for large data set, the DVM classifier would spend relatively more time in finding the representative vector, so multi-dimensional indexing techniques can be adopted to speed up search process to a certain extent.

### Procedure of proposed method

**Figure 9:**
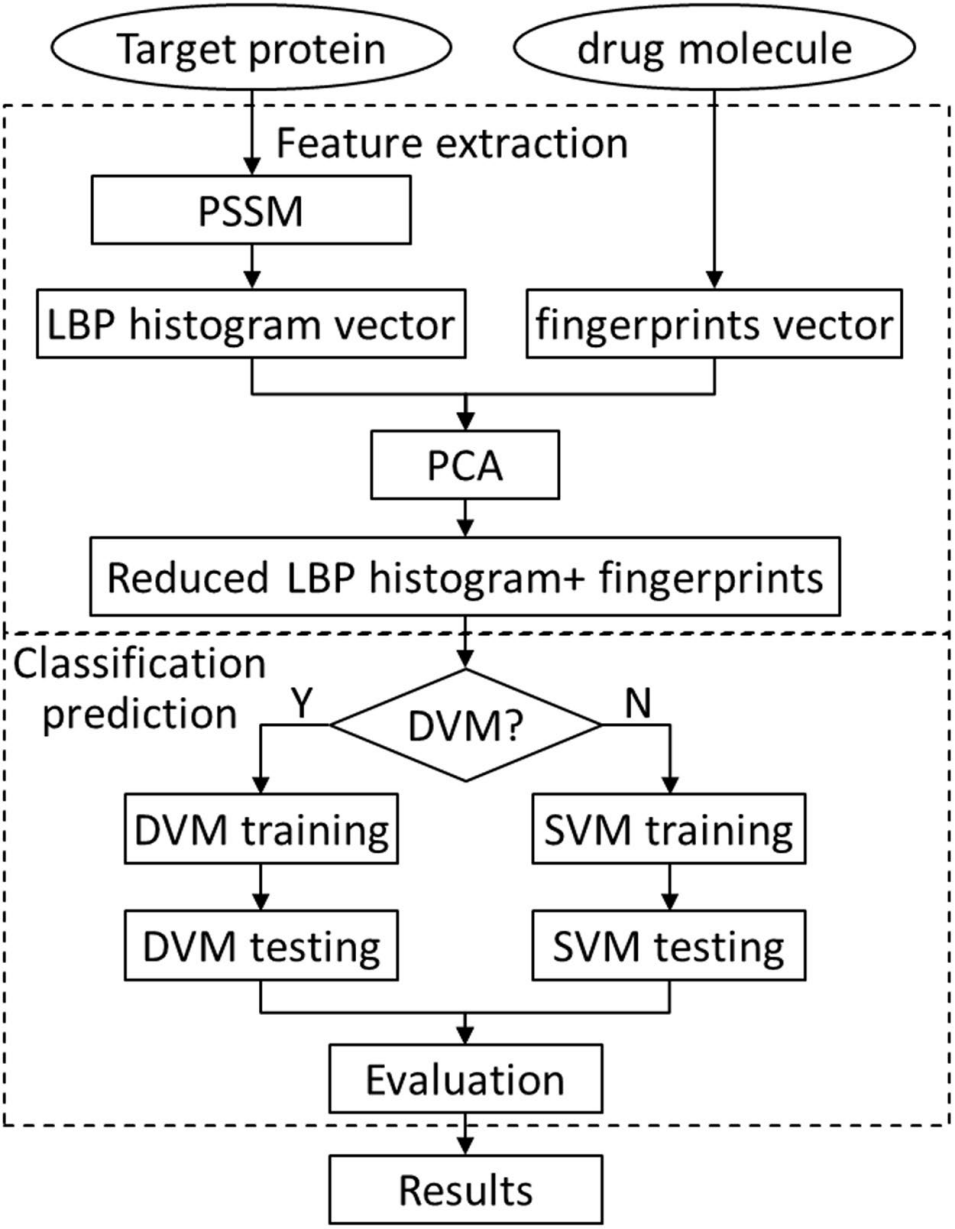
Flow chart of the proposed method.

In this work, the procedure of our proposed method mainly consists of two steps: feature extraction and classification prediction. The feature extraction also contains two sub steps: (1) the PSI-BLAST tool is employed to represent each target protein sequence and the corresponding PSSM is obtained; then LBP operator is used to obtain LBP histogram vector. (2) Based on substructure information of drug molecule, the fingerprint vector of drug molecule is calculated. Then the corresponding DTI pair is constructed by concatenating the two vectors of protein sequence and drug substructure. To reduce the computational burden and suppress the effect of noise, principal component analysis (PCA) method is then employed to extract the highly discriminatory feature information. As mentioned before, each of the four datasets (*Enzyme*, *GPCR*, *Ion Channel and Nuclear Receptor*) is divided into training set and test set separately. Then the classification prediction on each dataset is also divided into two sub-procedures. (1) The training set is used to train the DVM model; (2) the trained DVM model is employed to predict DTIs on the four datasets and the performance metrics are evaluated correspondingly. In the same way, the SVM model is also built for predicting DTIs on these *four* datasets. The flow chart of our approach is shown as Figure 9.
